# Role of a Deep‐Learning Based Convolutional Neural Network Model for Real‐Time Ventricular Tachycardia Alarm Classification

**DOI:** 10.1111/jce.70271

**Published:** 2026-01-26

**Authors:** Unmesh Khanolkar, Ashish Yadav, Avdhesh Mann, Scott Pappada, Abhishek Deshmukh, Abhishek Maan

**Affiliations:** ^1^ Division of Cardiac Electrophysiology University of Toledo Toledo Ohio USA; ^2^ NYU Tandon School of Engineering New York New York USA; ^3^ Indian Institute of Technology Mandi India; ^4^ Department of Bioengineering University of Toledo Toledo Ohio USA; ^5^ Department of Anaesthesia University of Toledo Toledo Ohio USA; ^6^ Mayo Clinic Rochester Minnesota USA

**Keywords:** alarm fatigue, convolutional neural network, deep learning, ventricular tachycardia

## Abstract

**Background:**

Ventricular tachycardia (VT) is a life‐threatening arrhythmia that requires both relatively rapid and accurate detection in intensive care units (ICUs). Continuous monitoring systems play a crucial role in detecting them. However, previous studies have reported that nearly 9 out of 10 arrhythmia alarms in ICUs tend to be false positives, which usually transpire to a well‐documented phenomenon called “alarm fatigue” that leads to desensitization, delayed responses, and increased cognitive burden on healthcare providers.

**Methods:**

We developed a deep learning based, one‐dimensional convolutional neural network (1D‐CNN) to classify VT alarms using multiple raw waveform inputs, including two electrocardiogram (ECG) leads, photoplethysmogram (PPG) and arterial blood pressure (ABP) signals. The model was trained using the publicly available VTaC Arrhythmia Benchmark Dataset. We used the 10‐second waveform segments that preceded each VT alarm, pre‐processed and then used them to train the machine learning model to correctly classify the VT alarm.

**Results:**

On the test set, the model achieved an area under the receiver operating characteristic curve of 0.901, overall accuracy of 83.22%, F1‐score of 73.3%, sensitivity of 77.53%, specificity of 85.63%, and positive predictive value of 69.57%. The model successfully detected over three‐quarters of them while significantly reducing false positive rates for the detection of VT.

**Conclusions:**

This study demonstrates that a deep learning based 1D‐CNN model using short segments of raw waveform data can achieve robust performance in distinguishing true and false VT alarms.

## Graphical abstract

Our manuscript discusses the role of a Convolutional Neural network based Deep‐learning model for the classification of ventricular tachycardia alarms. Our algorithm based on input from multiple raw waveform inputs, including electrocardiogram leads, photoplethysmogram, and arterial blood pressure signals achieved a good degree of accuracy and performed well in terms of sensitivity and specificity

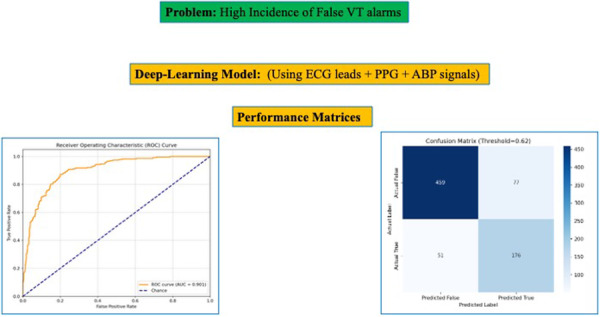

**List of abbreviations**
AUC‐ROC: area under the receiver operating characteristic curve
ABP: arterial blood pressure
ABG: arterial blood gas
ECG: electrocardiogram
ICU: intensive care unit
1D‐CNN: one‐dimensional convolutional neural network
PPG: photoplethysmogram
PPV: positive predictive value
ROC: receiver operating characteristic
PLETH: plethysmogram
VT: ventricular tachycardia

## 1 Introduction

Ventricular tachycardia (VT) is a life‐threatening arrhythmia that requires rapid identification and intervention in intensive care units (ICUs) as well as in hospitalized patients [1]. Continuous monitoring systems play a crucial role in detecting such events, but their performance is hampered by the persistent problem of false alarms [2]. The issue of false alarms typically stems from the limitations of the currently available algorithms that adjudicate VT and other arrhythmias. These false alarms tend to be a major driver for the phenomenon of alarm fatigue. Alarm fatigue is a state in which repeated exposure to false alarms leads to desensitization and delayed or missed responses to true clinical events [3]. This phenomenon is a significant patient safety concern as well as a clinical inaction during true events of VT. Both these findings of alarm fatigue and clinical action are well‐described in the current published literature. Based on previous studies, that nearly 90% of the arrhythmia alarms in ICUs are false positive alarms [4, 5]. The rates of inaccuracy are even higher regarding the occurrence of VTs [5]. These high alarm burdens divert attention and lead to cognitive overload in health‐care personnel working in ICUs [3]. Previous approaches have used manual features to train the model, and considering that these manual features depend on predefined characteristics and therefore might overlook subtle yet clinically relevant waveform features. Deep learning (DL) allows the model to learn directly from the raw waveform data, thus capturing the complex features that might otherwise get omitted in other machine learning models, especially those that entail manual annotation [6, 7].

In our study, we developed a DL‐based, one‐dimensional convolutional neural network (1D‐CNN) model to further classify VT alarms using multiple raw waveform inputs. As the input data source, we used a publicly available VTaC Arrhythmia Benchmark Dataset hosted over physionet (https://physionet.org/). The major aim of our study was to develop a DL‐based model that could be capable of reducing false positives while maintaining high sensitivity, with the final goal of mitigating the issue of alarm fatigue.

## 2 Methods

### 2.1 Dataset

This machine learning algorithm was created using the VTaC Arrhythmia Benchmark Dataset (https://physionet.org/physiobank/database/vtac2012/). This is a publicly available dataset hosted on PhysioNet, and is a relatively rich resource of multi‐channel physiological recordings focused on VT alarms [8, 9]. For the purpose of maintaining a diversified and clinically representative sample in our study, we used the patient‐recorded data from a total of three large commercial US vendors, as well as the data from the ICUs of three major U.S. hospitals. This study was exempt from Institutional Review Board approval, considering the publicly available and de‐identified nature of the dataset used.

There are 5037 annotated VT alarm occurrences in the dataset, which are derived from a total of 2376 distinct patients. Each recording in the dataset consists of a 10‐min segment which encompasses the onset of VT alarm and has data of 5 min of waveform before the alarm and 5 min after the occurrence of VT. For the purpose of maintaining diversity in terms of data collection, at least 5 alarm events from an individual patient were selected. The annotation process of alerts was performed by professional experts who were randomly assigned batches of VT. In addition, the overall annotation team comprised: an expert physician, highly experienced board‐certified cardiac arrhythmia technician, 3 other clinicians, and 1 biomedical signal processing engineer with extensive expertise in cardiac arrhythmias. Through this process, 1441 alerts (22.8%) were classified as “True” VT occurrences. Each recording includes electrocardiogram (ECG) leads and one or more pulsative waveforms, such as arterial blood pressure (ABP) and photoplethysmogram (PPG/PLETH). The signals were uniformly resampled at 250 Hz.

### 2.2 Data Splitting

The list was initially shuffled at random to guarantee a fair distribution of patients among the dataset splits. A fixed seed value of 42 was used for this randomization, ensuring that the data partitioning procedure is repeatable across runs and environments. After data shuffling, the patient list was divided into three exclusive sets based on a 70/15/15 ratio. The alarm events associated with the patients in each set were then allocated to their respective training, testing, and validation sets. The goal of this allocation was to enable the DL‐model to accurately determine expected performance in a realistic clinical setting, such as the one based on a sample of novel patients.

### 2.3 Signal Pre‐Processing

We processed a total of four channels of signals: two ECG leads, a photoplethysmogram (PLETH) signal, and an arterial blood pressure (ABP) waveform. A 10 s window of data, which consists of 2500 data points at the original 250 Hz sampling rate, was taken from a segment preceding each arrhythmia alarm. This segment was chosen specifically as it would most likely contain the arrhythmic patterns that occur immediately prior to the arrhythmia alarm. However, when we noticed the data segments with missing or non‐finite values, we addressed these by replacing them with a value of zero. This also allowed for numerical stability, which is required for the subsequent stages of filtering [10].

Once the data segments were stabilized, we applied a series of filter specific to each of the 3 channels to further refine the data and also to diminish signal‐related noise. To understand the noise in the VTaC dataset, we began by examining the power spectrum across all ECG channels. This check revealed that 60 Hz power line interference was present in all the ECG tracing across all channels. We noticed a clear peak in all signals, and the average signal‐to‐noise ratio was 6.8 dB. First, a 60 Hz notch filter was applied across all channels to remove the power‐line interference. Following this, filtering was applied to each type of signal to isolate the most informative frequency bands. The ECG signals were passed through 1.0–3.0 Hz band‐pass filter, which removes the low‐frequency baseline wander and high‐frequency myopotentials while still preserving the critical components of the cardiac cycle. The PLETH signal was treated with a 0.5–5.0 Hz band‐pass filter, and the ABP signal was refined with a 16 Hz low‐pass filter.

**Figure 1:**
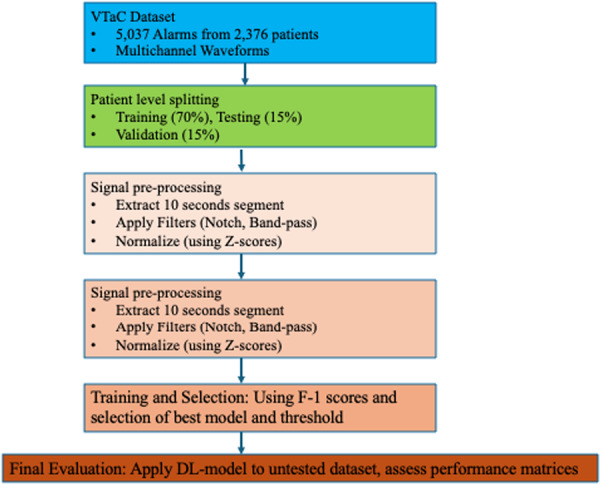
Schema describing our study design.

The final step was to address the issue of variation in the signal amplitudes between patients and recordings. We used sample‐wise z‐score normalization to standardize each patient segment, which serves to remove inter‐patient amplitude variation and emphasizes signal morphology. This approach has been shown to generalize well across heterogeneous populations, as it reduces the need for population‐specific calibration [11]. This normalization technique is aimed at centering the signal at a mean of zero with a unit standard deviation. This also assists the deep‐learning model to further focus predominantly on the shape and pattern of these waveforms. Our study design is summarized in Figure 1.

### 2.4 Model Architecture

We developed a deep‐learning model based on one‐dimensional CNN architecture to classify binary classes of “true” VT alarms and “false” VT alarms. Our DL model architecture consists of four successive convolution blocks that serve as feature extractors. A network block holds two sequential 1D convolutional layers. Batch Normalization and a ReLU activation function follow each layer. The layers extract the temporal features from the input signal. A Max‐Pooling layer then cuts the temporal resolution at the block's end. This shortens the time dimension as well as controls the computing needs. The filter count grows with the network's depth, with 128 filters in the first block, and more filters in later blocks—this allows the model to learn further complexity.

**Figure 2:**
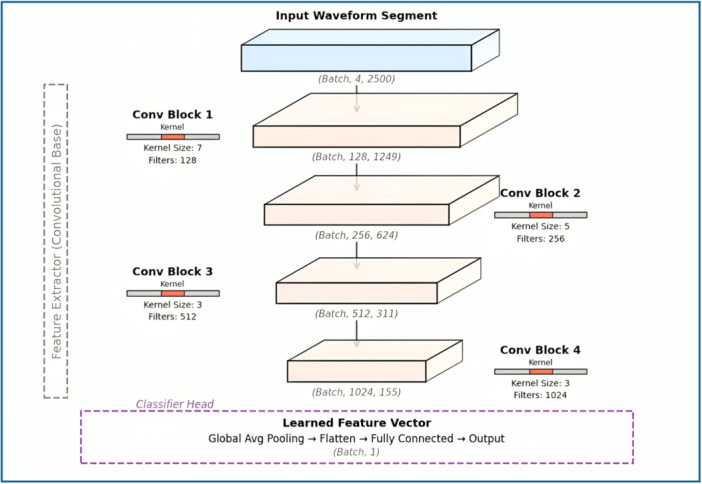
Detailed architecture of the deep‐learning model in our study.

To aggregate the features from the convolutional blocks in the final step of our DL‐algorithm, we then used a global average pooling layer. The layer compresses the temporal dimension of the final feature maps into a fixed‐length vector and has also been previously described to simplify the interpretation of DL algorithms and prediction. This vector is then inputted into a completely connected classification head of a dense layer with 256 units, ReLU activation, and a 50% dropout layer to prevent overfitting. The final output is a single logit for binary classification. The summary of model architecture used in our study is shown in Figure 2.

### 2.5 Feature Extraction

In contrast from the previously published machine‐learning models that have relied on manual definition and annotations [12, 13]. Our study used a deep‐learning model for the classification of VT alarms. An approach based on deep learning has the advantage of recognizing and capturing features that are challenging to be perceived using the existing rules. Furthermore, an unsupervised learning method also has the advantage of being less time‐consuming and resource‐intensive as compared to supervised learning method. In our study, we used a unidimensional, Convolutional Neural Network as the unsupervised learning method. The structure of our DL‐based, 1‐dimensional CNN model, along with its function of feature‐extraction from the existing data, is summarized in Table 1.

**Table 1:** Features of the deep‐learning‐based convolutional neural network model.

Layer	Description	Output shape (Batch, Channels, Length)	Parameters/Configuration
Input	Time‐series segment with 4 channels.	(B, 4, 2500)	segment_length: 2500, input_channels: 4
Conv Block 1	2x [Conv1d, BatchNorm, ReLU], 1x MaxPool1d	(B, 128, 1249)	base_filters (K): 128, F: 7, Pool: 3
Conv Block 2	2x [Conv1d, BatchNorm, ReLU], 1x MaxPool1d	(B, 256, 624)	Filters: 2K (256), F: 5, Pool: 3
Conv Block 3	2x [Conv1d, BatchNorm, ReLU], 1x MaxPool1d	(B, 512, 311)	Filters: 4K (512), F: 3, Pool: 3
Conv Block 4	2x [Conv1d, BatchNorm, ReLU], 1x MaxPool1d	(B, 1024, 155)	Filters: 8K (1024), F: 3, Pool: 3
Global Avg Pooling	Aggregates features across the time dimension.	(B, 1024, 1)	—
Flatten	Flattens feature maps for the dense layer.	(B, 1024)	—
Fully Connected	Dense + BatchNorm + ReLU + Dropout	(B, 256)	W₁, b₁, Dropout: 0.5
Output Layer	Single Linear neuron for binary classification.	(B, 1)	W₂, b₂ (Outputs raw logits)

The steps used for building the DL‐based, unidimensional CNN model in our study also circumvented the need for manual annotation of features. Furthermore, given the time‐series‐based nature of the data on cardiac rhythms, the DL model learns further patterns in an unsupervised manner [14]. The different layers used in the network filter the data further and help find the relevant information in an automated manner. The network advances the input signal through a separate four layers of convolutional blocks. In the previous layers, such as the **Conv Block 1**, the kernel sizes are generally larger (F: 7). Here, the network detects simple patterns such as steep slopes, outlines, and simple components of waveforms. In the further deeper layers of the network, such as **Conv Block 3** and **4**, although the kernel sizes are much smaller, the number of filters is vastly increased to the extent of 1024. Through these CNN blocks, the model's network first utilizes the simpler patterns, which are in the shallow layers, and then incorporated them into the features that are more complex and abstract. These features are representative of the overall shapes, such as the relationships between ECG and ABP waveforms, or lack thereof.

At the final convolutional layer, the raw input signal which was originally shaped as (B, 4, 2500) was subsequently transformed into a high‐dimensional feature map with shape (B, 1024, 155). This resulting tensor represents the learned features which are extracted by the model, capturing important patterns from the input signal that are relevant for classification of VT events as true versus false VT alarms.

### 2.6 Training and Evaluation

The model was trained using a Binary Cross‐Entropy with Logits loss function, which is a common option for binary classification tasks and can improve numerical stability [15]. One of the significant challenges in our dataset was the apparent class imbalance of “false alarms” significantly outnumbering “true alarms.” To address this limitation, we initially employed a loss‐weighting approach that is designed to prevent the model from unintended biases towards the majority class.

The approach consists of calculating a weight for the positive class as the ratio of negative samples to positive samples from the training data and multiplying the loss of the positive class (true alarms) by that weight. In effect, if the model misclassifies a true alarm to the negative class, the loss is multiplied by the positive class as defined above. This scales the larger error signal during the backward pass and forces the model to update its parameters more robustly to identify the features of the infrequent, but critical positive class.

One of the advantages of this approach is that it is computationally efficient, and it utilizes the entire data without resampling and losing the distribution of the data, while still being able to achieve its goal. On the contrary, such an approach also had its own limitations. For instance, by enabling the model to be relatively more sensitive to the minority class often leads to a diminution of specificity. This would subsequently contribute to an increase in the false positive rates. Ideally, we aim to enhance the model's sensitivity (recall), ensuring that it can reliably detect true arrhythmic events. At the same time, it is equally important to preserve a high level of specificity, which means accurately identifying false alarms to avoid unnecessary clinical interventions.

The model weights were updated using the AdamW optimizer and were selected as a natural fit for its versatility in addressing the weight decay, with a learning rate of 1 × 10⁻⁴ that also includes a definition for weight decay being 0.01 in order to regularize the model and prevent overfitting in several iterations. The model was trained for 100 epochs with training monitored using an early stopping criterion. This approach ensures that training would halt automatically if the model's performance on the validation could not improve any further, helping the model to prevent overfitting. If the model's F1 score on the validation set did not result in any improvement for 30 epochs, then the model would conclude the training process.

The model determined for selection was the checkpoint with the highest F1 score in the validation set. The final method required to move from the continuous probability output to a predicted classifier was to determine the optimal classification threshold. The optimal threshold was determined by selecting the threshold that would maximize the F1 score when evaluated on the predictions from the validation set. The optimal threshold was then applied to the test set predicted probabilities to derive the final performance metrics. The code of our deep‐learning model is made available based on a reasonable request.

### 2.7 Class Imbalance

Considering that the overall occurrence of VT is overall infrequent, as compared to sinus rhythm. We also acknowledge that this introduced the issue of class‐imbalance, which is encountered frequently in the application of ML algorithms for the detection of cardiac arrhythmias. To address this issue, the SMOTE approach, which aims at oversampling of infrequent events, has been utilized [16]. This approach could necessitate the creation of new data as opposed to the duplication of minority events for the enrichment of dataset. Previously published studies have also utilized the Focal Loss model to address the issue of class imbalance. This approach focuses on the minor set of events (such as cardiac arrhythmias) by increasing their relative importance and makes the ML model more applicable to the minority class of events (i.e., cardiac arrhythmias) [17]. Although it is important to note that an important limitation of such an approach is the increased computational cost. In line with these approaches, for our dataset, we compared both the SMOTE (as a data‐level approach) and the Focal Loss model approach and observed that the latter approach was more suitable for our analyses. The comparison of performance metrics using these 2 approaches is summarized in Table 3.

### 2.8 Performance Metrics

We evaluated the model's performance on the test set using standard classification metrics, which were calculated after applying an optimal classification threshold determined on the validation set. The model's ability to correctly identify true ventricular tachycardia (VT) alarms was measured by its *sensitivity (recall)*, while its capacity to correctly dismiss false alarms was assessed by its *specificity*. We also calculated the precision, or positive predictive value, which represents the proportion of alarms classified as true and were indeed true VT events. To provide a balanced measure of performance, particularly given the class imbalance inherent in the dataset, we computed the F1‐score, which is the harmonic mean of precision and sensitivity. The F1‐score is a measure that accounts for both the precision and sensitivity of the deep‐learning model. It does so by penalizing the model if either of these measures is low. As the final evaluation of performance metrics, we also calculated the overall accuracy of the model.

We also computed the Area Under the Receiver Operating Characteristic Curve (AUC‐ROC) in addition to these threshold‐dependent metrics. The ROC curve plots Sensitivity (True Positive Rate) against 1 ‐ Specificity (False Positive Rate), providing a comprehensive view of the trade‐off between true positive and false positive rates. A perfect classifier is represented by an AUC‐ROC of 1.0, whereas performance equal to random chance is represented by an AUC‐ROC of 0.5. These metrics were computed by computer programming using the *scikit‐*learn library in Python. The final performance report on the test set utilized Accuracy, Precision, Recall, Specificity, F1‐score, and AUC‐ROC.

## 3 Results

**Figure 3:**
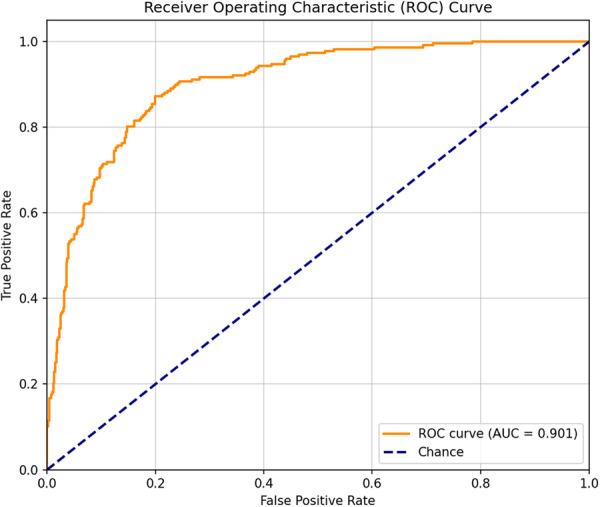
Receiver Operating Characteristic (ROC) Curve. The model's classification performance on the test set at all thresholds is illustrated by the curve, resulting in an Area Under the Curve (AUC) of 0.901.

**Figure 4:**
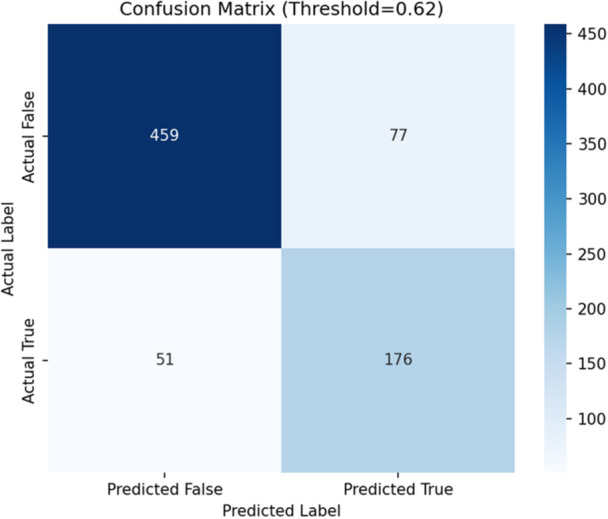
Confusion matrix. Classification performance on the test set at the optimal decision threshold of 0.62.

In our study, the one‐dimensional, deep learning‐based, CNN model performed well on an unseen training dataset when evaluated for differentiating true versus false alarms for VT. Our model also demonstrated a high degree of efficiency when classifying the VT alarms. The model was able to achieve an Area under the Receiver Operating Characteristic value of (AUC_ROC) 0.901 as shown in Figure 3. The value of 0.901 illustrates that the model also has a good ability to rank a true‐alarm higher than a false‐alarm. These findings imply that a deep learning architecture based on a combination of waveforms can learn the subtle distinctions between the events of true versus false VT alarms. We also evaluated the performance of our model using several threshold‐dependent metrics in addition to the AUC‐ROC. These metrics provide further insights into how well the model performs at a specific decision point, which is critical for its deployment in a real‐world setting. An optimal threshold value of 0.62 was established by maximizing the F1‐score on the validation set predictions. When this chosen threshold was applied on the test set, the results were as seen in the confusion matrix presented in Figure 4. The model was able to correctly identify 176 of the 227 true VT alarms (True Positives) and correctly dismiss 459 of the 536 false alarms (False Positives).

As shown in Table 2, the model was able to achieve an overall accuracy of 83.22% and a balanced F1‐score of 73.3%. The recall or sensitivity value of 77.53% is a critical marker as it signifies that the model can successfully detect over three‐quarters of the VT events (and the clinical relevance of this is its ability to classify an arrhythmia that can result in sudden cardiac death). The specificity value of 85.63% implies that the model has the potential to mitigate the issue of alarm fatigue, which is common with the current models. The precision or Positive Predictive Value of 69.57% indicates that the model is correct nearly 70% of the time when it encounters a true VT alarm. This reflects a substantial improvement, as only 28.65% of the total alarms in the VTaC: A Benchmark Dataset of Ventricular Tachycardia Alarms from ICU Monitors dataset were consistent with true VT events. We also utilized Focal loss model and SMOTE model in our ML analysis, and these results are summarized in Table 3.

**Table 2:** Final model performance metrics on the test set.

Metric	Value
AUC‐ROC	0.901
F1‐Score	0.733
Accuracy	0.832
Recall (Sensitivity)	0.775
Precision (PPV)	0.696

**Table 3:** Use of Focal Loss and SMOTE model to address class‐imbalance and performance matrices of our ML model.

Performance metric	Focal Loss model	SMOTE model
Sensitivity	96.0%	92.5%
Specificity	58.6%	51.5%
AUC	0.902	0.868

On balance, we also acknowledge the limitations of our model. As shown in the confusion‐matrix, there were indeed a total of 51 instances of misclassification of a true VT alarm. We suspect that these could be due to challenging physiological patterns or due to the presence of artifacts in the signals collected. The model also yielded a total of 77 false positives where a non‐VT alarm was classified as a true VT episode. We speculate that these could be attributed to the presence of significant signal artifacts (e.g., from patient movement, myopotentials, or misplacement of EKG electrodes), which can still present challenges for a deep‐learning model such as ours. Our observation validates the use of a DL approach for the classification of clinical arrhythmias. Although our analysis was derived from a 10‐second, raw, multi‐channel waveform data preceding an arrhythmia alarm. But our 1D‐CNN model can still provide a relatively strong tool for automated VT alarm classification. The model was also able to achieve a strong balance between sensitivity to critical events and offering the advantage of being able to decrease the rate of false‐alarms.

## 4 Discussion

The major findings from our study are as follows:

1. We developed a DL‐based model to address the current issue of alarm fatigue in the ICU settings.
2. Our model is a one‐dimensional‐CNN based, that automatically classifies VT alarms using three physiologic waveforms‐ ECG, ABP, and PPG.
3. Our model utilized the VTaC Arrhythmia benchmark dataset, which has a total of 5037 annotated alarms.
4. Our DL‐based model successfully achieved an AUC‐ROC value of 0.90 with an overall accuracy of 83.22%. The specificity of 85.63% achieved in our model attests to a significant reduction in the rate of false‐positive annotation of VT events.
5. The precision of 69.67% is also significant, given the class imbalance due to true VT events consisting of only 28.6% of all alarms. These results suggest that our model has the potential to ease off alarm fatigue and reduce the burden on healthcare providers.

The performance of our model emphasizes the relative importance of a DL‐based approach as compared to the previously used approaches. Conventional ML‐based models for the detection of cardiac arrhythmias predominantly rely on the manually extracted features from the waveforms. Considering this reliance on manual extraction of features, these conventional ML‐based models can have limitations that are inherent to human bias as well as due to an incomplete representation of features. On the other hand, our Uni‐dimensional‐CNN model self‐learns from the raw waveform data, thus enabling it to capture fine patterns that may distinguish true VT alarm from a false VT alarm. Besides that, given the multi‐modal nature of our model (considering that it incorporates data from 2 ECG leads, PPG, and ABP waveforms), it has a superior diagnostic profile for differentiation of VT alarms. Mechanistically, our deep‐learning, multimodal approach, which incorporates data from the ECG leads, PPG, and ABP, is more robust than a traditional, supervised ML model. The multimodal and deep‐learning aspects of this also enable our model to account for the electrical and mechanical fingerprints of an episode of VT.

The reduction of false alarms is particularly relevant in the context of ICU alarm fatigue. In a previously published study by Drew et al., based on a total of 31 ICU units that had evaluated 12,671 annotated arrhythmia and demonstrated that up to 88.8% of the total arrhythmia alarms were false positives [4]. Similarly, in other study by Harris et al, the overall false‐positive rate for ventricular tachycardia alarms was 86.8%. These investigators speculated that factors such as bundle branch block, ventricular pacing, mechanical ventilation, and patient agitation were the dominant contributors to the inaccuracy of arrhythmia alarms [6]. These significantly high rates of false alarms not only desensitize the staff but also divert their attention from the alarms that indeed represent a true VT episode. By achieving a specificity above 85%, our model could help mitigate the burden of these false‐alarms. At the same time, a sensitivity of 78% represents an acceptable trade‐off.

Besides its performance for improving the accuracy of arrhythmia alarms, our DL‐based approach also offers operational advantages. Our model only requires 10 s of waveform data prior to the alarm for VT. This relatively shorter duration could enable us to potentially implement our model in a near‐real‐time manner in the routine bedside monitors. The shorter waveform analysis window also reduces the overall computational load, thus making deployment more feasible in resource‐constrained settings. Besides that, we used loss weighting instead of oversampling to address the issue of class imbalance. This adjustment preserved the natural class distribution, thus reducing the risk of overfitting and maintaining the generalizability of our model.

### 4.1 Comparison with Current Studies

A few previous studies have also utilized ML algorithms to differentiate cardiac arrhythmias. For instance, a study by Lin Q. et al. had utilized the techniques of similarity maps and feature extraction from the learnable Parzen band‐pass filters. This approach captures similarities amongst different segments of the EKG tracing. In this study, the investigators described an overall high degree of sensitivity for the detection of VT and ventricular fibrillation [18]. Similarly, Zhou et al. used a deep‐contrastive learning framework to determine “true” arrhythmias with the final goal of mitigating false alarms. In their study, the investigators had used the framework of CNN as signal encoders to reduce the length of input signals from the multi‐waveform data and had also used pairwise loss of function to address class imbalance to mitigate the low event rates of cardiac arrhythmias [19]. In another study by Afghah et al., the investigators had used a predictive modeling technique based on the coalition game therapy, which incorporates inter‐feature dependency to improve net classification of arrhythmias [20]. In their subsequent study, the same investigators also used an unsupervised feature extraction from 5‐min‐long recordings to develop an unsupervised feature learning model, which was based on unannotated EKG signals as input. The input was then clustered to assess linear and non‐linear relationships with the final goal of differentiating true versus false alarms [21]. However, in comparison to our study, the overall number of alarms analyzed in this study was relatively low and their final assessment achieved an AUC of 0.85.

In conclusion, our DL‐based uni‐dimensional CNN model demonstrates strong performance in classifying VT alarms using short segments of raw waveform data from multiple inputs (EKG, PPG, and blood pressure monitoring). By striking a balance between sensitivity and specificity, this approach has the potential to alleviate alarm fatigue and hence improve patient safety in ICU settings. In future, prospective studies that could integrate our model into the clinical decision support system could help in the validation of our model. Model interpretability techniques such as saliency mapping could provide physicians with a visual explanation of alarm classification, which would help in its further adoption in routine clinical practice [22]. In addition, the use of saliency mapping could be particularly helpful in a model such as ours, given the time‐series nature of rhythm changes and would also help assess the role of individual features of cardiac arrhythmias from ECGs and rhythm tracings.

### 4.2 Limitations

We acknowledge several limitations of our study. First, although the VTaC dataset is diverse, it is limited to three U.S. hospitals, and external validation on diverse monitoring systems is necessary to confirm generalizability. Second, our preprocessing pipeline standardized the signals and then applied filters. But the real‐world ICU signals might contain more variability, such as sudden changes in the lead placement, or nonstationary noise, both might further contribute to false‐positives. Third, we also acknowledge that our deep‐learning model was trained on retrospective data, and its prospective evaluation in a live ICU environment would be necessary for its further validation. In addition, we also acknowledge that we used a relatively simpler imputation strategy for handling of missing or discontinuous data. Our rationale of using a simpler imputation strategy was based on the goal of keeping the model numerically stable. The model's performance could likely be enhanced by using more advanced imputation techniques, like linear interpolation or forward‐filling for short gaps or even employing generative models to reconstruct longer missing segments.

## 5 Relationship to Industry

**AM:** Consulting: Biotronik, Vektor Medical. Other authors have no relevant disclosures.

## Funding

The authors received no specific funding for this work.
